# Two-Dimensional Chromatographic Isolation of High Purity Erinacine A from *Hericium erinaceus*

**DOI:** 10.3390/jof11020150

**Published:** 2025-02-15

**Authors:** Katerina Naumoska, Andrej Gregori, Alen Albreht

**Affiliations:** 1 Laboratory for Food Chemistry, Department of Analytical Chemistry, National Institute of Chemistry, Hajdrihova ulica 19, 1001 Ljubljana, Slovenia; katerina.naumoska@ki.si; 2 Mycomedica Ltd., Podkoren 72, 4280 Kranjska Gora, Slovenia; andrej.gregori@zanaravo.com; 3 Biotechnical Faculty, University of Ljubljana, Jamnikarjeva ulica 101, 1000 Ljubljana, Slovenia

**Keywords:** erinacine A, *Hericium erinaceus*, food supplement, 2D heart-cut chromatography, isolate, standard

## Abstract

A simple and robust two-dimensional chromatographic fractionation protocol for the isolation of the neuroprotective compound erinacine A from *Hericium erinaceus* is proposed. This production platform yielded 19.4 mg of erinacine A from approximately 130 g of mushroom material, with a chromatographic purity of 97.4%. The procedure includes extraction, concentration, fractionation, purification, and characterisation of the bioactive compound. The crude *H. erinaceus* extract was fractionated in the first dimension by normal-phase flash chromatography, and the fraction containing erinacine A was further purified in the second dimension by semi-preparative reversed-phase chromatography. This strategy utilises the orthogonality of the two chromatographic modes to effectively eliminate difficult impurities, including structural isomers and analogues of erinacine A. Complementary analytical approaches such as high-performance thin-layer chromatography (HPTLC) and high-performance liquid chromatography with ultraviolet and tandem mass spectrometric detection (HPLC–UV–MS/MS) were employed to unambiguously confirm erinacine A in the isolated fractions, while HPLC with a charged aerosol detector (CAD) was used to determine its purity. Given the limited commercial availability and the high price of erinacine A, the described method offers a straightforward and cost-effective alternative to obtain this valuable compound for further research and applications.

## 1. Introduction

Medicinal mushrooms, which have been used for centuries in traditional Chinese medicine, offer a viable alternative to modern medicine by providing effective solutions for diseases that cannot be treated with conventional therapies [1]. *Hericium erinaceus* (Bull.: Fr.) Pers., commonly known as Lion’s Mane, is a well-known medicinal mushroom that has attracted great interest from academia and the industry as it contains a wide range of health-promoting compounds, with erinacine A being particularly noteworthy [2,3,4]. Erinacine A is a diterpenoid that is valued for its strong neuroprotective properties. It stimulates the synthesis of nerve growth factor, thereby improving cognitive function [5]. This property makes erinacine A a valid therapeutic agent for neurodegenerative diseases [6], such as Alzheimer’s, Parkinson’s, Huntington’s disease, and multiple sclerosis. In addition, erinacine A has been shown to reduce the deposition of β-amyloid and increase the expression of the gene encoding the insulin-degrading enzyme [7], further supporting its potential in combating neurodegeneration. Neurodegenerative diseases affect millions of people worldwide and have an impact on patients, families, and carers. Although there is currently no cure, therapies focus on alleviating symptoms and delaying disease progression to improve quality of life. Ongoing studies are focussing on the underlying disease mechanisms and the corresponding key chemical species to understand and propose new effective treatments [8].

The production of erinacine A in *H. erinaceus* typically involves submerged fermentation, but many cultivation parameters influence the yield. Considerable variability in erinacine A content has been observed among different strains, with wild strains often showing better results [9]. Scientific advances in the field are enabling better yields of erinacine A, with submerged cultivation techniques showing good potential to increase erinacine A production [10]. In addition to submerged cultivation, solid-state cultivation has also been explored. The research indicates that solid-state fermentation can significantly increase the production of erinacine A under carefully selected conditions and provide higher levels of the compound compared to submerged methods. Accumulation of erinacine A can be improved by selecting suitable substrates and optimising growth conditions [11]. These advances suggest that both submerged and solid-state cultivation offer great potential for the further development of industrial production of erinacine A, thus supporting the continuity of research for this therapeutic. Corana et al., 2019 discuss various metabolites in *H. erinaceus*, including erinacine A, which is predominantly found in the mycelium. The study highlights the variation in bioactive compound levels across different fungal growth stages, such as mycelium, primordium, and sporophore. It emphasises the need for optimised cultivation conditions to increase metabolite production [12]. Tachabenjarong et al., 2023 emphasise the importance of choosing the right harvest period to maximise erinacine A content and its therapeutic properties [13]. Therefore, simple monitoring methods that allow the determination of the period when the accumulation of erinacine A in *H. erinaceus* is at its highest are crucial for large-scale production.

Obtaining a sufficient quantity of pure erinacine A is often of interest as it facilitates further clinical trials and the development of new, safe, and effective treatment strategies, paving the way for novel therapeutic products. This requires a simple, robust, and reproducible methodology for the production of erinacine A on a larger scale. However, selectivity and efficiency of production remain a challenge due to (i) a heavy fungal matrix, (ii) interference from structurally similar erinacines [8,14], and (iii) variability in the biosynthetic yield of erinacine A between different strains [9], which further complicates standardisation of the isolation process.

To date, only a few methods have been proposed for the isolation of erinacine A, and most are based on low-resolution, labour-intensive open-column chromatography, e.g., [10,15,16,17,18]. Advances in high-performance liquid chromatography (HPLC) and mass spectrometry (MS) have somewhat improved the reproducibility of targeted purification of compounds. Current purification strategies consist of multiple chromatographic and/or solid-phase extraction (SPE) steps to achieve a high degree of compound purity required for food and pharmaceutical applications. The optimisation of these methods is not only important to achieve high production yield and high purity of the isolate, but also to identify and monitor the bioactive compound with a higher degree of certainty throughout the isolation process. In addition, selective isolation of erinacine A in the presence of structurally related erinacines [8,14] poses a significant challenge, increasing the need for innovative extraction/isolation strategies that provide satisfactory results.

More recently, Liu et al., 2024 described a high-speed countercurrent chromatographic (HSCCC) method for the isolation of erinacine A from *H. erinaceus* [9]. The selection of a suitable two-phase solvent system (consisting of *n*-hexane, ethyl acetate, methanol, and water in a ratio of 4.5:5:4.5:5, *v/v/v/v*) proved to be crucial for the efficient separation of the desired diterpenoid from the mycelial matrix. Cheng et al. 2021 developed a complementary purification strategy using self-packed silica gel columns and semi-preparative HPLC–UV [11]. They investigated the solid-state cultivation of *H. erinaceus* for the production of erinacine A and showed that maize kernels are the most effective substrate. Factors such as the particle size of the substrate and the addition of nitrogen sources or inorganic salts were shown to significantly influence both the mycelial biomass and the specific yield of erinacine A. Optimisation of an ultrasound-assisted extraction method was also described, where aqueous ethanol was used as an efficient solvent for the extraction of erinacine A, together with several other chemical species of *H. erinaceus* [19].

In view of its great therapeutic potential, we wanted to develop a simple, robust, and reliable method for the isolation of erinacine A with high purity that meets the stringent requirements of clinical application. In this framework, we utilised a two-dimensional fractionation by combining normal-phase (NP) and reversed-phase (RP) chromatography. By optimising the isolation procedure through the selection of the most suitable extraction solvent and enhancing the final isolate purity through chromatographic method optimisation, this study enables the potential application of erinacine A in nutraceutical and pharmaceutical products.

## 2. Materials and Methods

### 2.1. Chemicals and Materials

Ethanol (absolute anhydrous) was purchased from Carlo Erba (Val de Reuil, France), while ethyl acetate and formic acid (98–100%) were acquired from Supelco/Merck (Darmstadt, Germany). Acetonitrile, both of HPLC and LC-MS grade, was obtained from J.T. Baker (Gliwice, Poland). Ultrapure water (18 MΩ·cm) was generated using a Milli-Q water purification system (Millipore, Bedford, MA, USA). The pulverised *H. erinaceus* used in this study was sourced from Mycomedica Ltd. (Podkoren, Slovenia), marketed as a food supplement under the brand name “GOBA^®^ *Hericium erinaceus*”. This material consists of a *H. erinaceus* biomass along with its extracellular secondary metabolites, combined with whole-grain starch, which served as the substrate. The material was dried and milled to a powder with an average particle size of less than 250 µm [20]. Custom-made erinacine A standard was a courtesy of Prof. Dr. Hirokazu Kawagishi from Shizuoka University, Japan.

### 2.2. Preliminary Testing

#### 2.2.1. Preliminary Extraction from the *H. erinaceus* Commercial Product and Selection of an Extraction Solvent by HPTLC Screening

Initially, four solvents and solvent mixtures were screened to identify the most optimal solvent for the extraction of erinacine A from *H. erinaceus* biomass powder: (1) ethanol, (2) ethyl acetate, (3) 70% aqueous ethanol, and (4) 70% aqueous ethyl acetate. Each extraction was performed at a ratio of 1:100 (*w/v*) (1 g per 100 mL) and was assisted by ultrasonication for 15 min. The samples were afterwards filtered through a 0.2 µm PTFE filter (Chromofil, Macherey-Nagel, Düren, Germany).

An HPTLC silica gel 60 plate (20 × 10 cm; Merck) was pre-developed with chloroform/methanol (1:1, *v/v*) and dried in an oven for 30 min at 110 °C. Extracts (10 µL) were applied as 8 mm bands, 10 mm from the bottom of the plate, using a Linomat 5 (Camag, Muttenz, Switzerland) operated by VisionCATS software 3.1 (Camag). Additionally, the custom-made erinacine A standard (25 µg mL^−1^ in 70% ethanol, 10 µL) was applied on the same plate. The plate was developed in a horizontal developing chamber (tank configuration) using 6 mL of ethyl acetate/acetonitrile (3:2, *v/v*). A preconditioning step using 10 mL of the same solvent lasted 10 min. The plate was developed to a height of 8 cm in approximately 12 min. For plate derivatization, an anisaldehyde detection reagent was prepared (glacial acetic acid 20 mL, methanol 170 mL, sulfuric acid 16 mL, and anisaldehyde 1 mL) [21]. The plate was dipped in the derivatization reagent using an Immersion Device III (Camag) for 2 s, dried with a hair dryer, and then heated at 110 °C for 2 min using a TLC Plate Heater III (Camag). Finally, the images of the plates were captured using a TLC Visualizer (Camag) at 366 nm (before derivatization) and at 254 nm and under white light (after derivatization).

#### 2.2.2. Isolation of Erinacine A-Containing Fraction by Preparative TLC

Preparative TLC (PLC) (silica gel 60 plate 20 × 10 cm; Merck) was used to accelerate the development of the erinacine A production procedure by enabling the collection of an initial mass of a fraction containing erinacine A, sufficient to develop the LC–UV–MS/MS method. This method was subsequently used to characterize the isolates after 1^st^D and 2^nd^D isolation. A volume of 900 µL of the 70% ethanol extract (prepared as described in Section 2.2.1.) was applied as a 180 mm band. The HPTLC method described above was also applied for fractionation. After development, the plate edges were derivatized with the anisaldehyde reagent, and the fraction of interest was scratched off from the underivatised plate part. The silica adsorbent scratched off from two PLC plates was pooled and suspended in ethanol. The resulting suspension was filtered through a 0.2 µm PTFE filter (Chromofil, Macherey-Nagel) and subjected to HPLC–UV–MS/MS analysis.

### 2.3. Extraction of Erinacine A from the H. erinaceus Commercial Product for Large-Scale Isolation

*H. erinaceus* powder (20 g) was divided into two equal parts and each was extracted by using 70% aqueous ethanol (1 L). The suspension was sonicated for 15 min and then stirred at room temperature in the dark for 3 days. The combined extracts were then vacuum-filtered through a blue-ribbon filter paper (Sartorius, Göttingen, Germany) and evaporated under reduced pressure at 40 °C (Rotavapor, Büchi, Switzerland) to dryness. The resulting dry extract was reconstituted in 70% aqueous ethanol (40 mL; final concentration 500 mg mL^−1^). The extract prepared in this manner was considered one extraction batch. In total, 6.5 batches (13 extractions × 10 g mushroom material = 130 g) were carried out.

### 2.4. First-Dimension (1^st^D) Fractionation of Erinacine A by Flash Chromatography

An isocratic method was run on the PuriFlash 5.250 system (Interchim, Montluçon, France), operated by IntersoftX software version 10.19.347.11 (Interchim), using puriFlash silica cartridges (high capacity silica, 25 g, 15 µm spherical particles with 60 Å pores; Interchim). The mobile phase consisted of ethyl acetate and acetonitrile (2:8, *v/v*) and the flow rate was set to 5 mL min^−1^. The overall run time was 20 min, and 3 mL of crude extract (as described in the previous section) was injected. Chromatograms were recorded using ELSD at 40 °C and UV at 340 nm, and absorption spectra were acquired in the range 200–800 nm. The collection of erinacine A fraction was triggered by a UV detector at a threshold of 10 mAU. Collected fractions from six consecutive analyses were pooled, the solvent was removed under a gentle stream of nitrogen, and the dry solid residue was then reconstituted in 2 mL of 70% aqueous ethanol. The entire extract was processed by carrying out 85 fractionation runs.

### 2.5. Analysis of Flash Chromatographic Fractions by HPTLC

Fractions #1–4 from the flash chromatography (10 µL) along with the erinacine A standard (25 µg mL^−1^ in 70% ethanol, 10 µL) were applied on HPTLC silica gel 60 plate as 8 mm bands, 10 mm from the bottom of the plate, using Linomat 5 (Camag), and were analyzed by the above HPTLC method.

### 2.6. Second-Dimension (2^nd^D) Fractionation of Erinacine A—Containing Fraction by Semi-Preparative HPLC

The concentrated fraction from the 1^st^D fractionation that was abundant in erinacine A was injected into the Infinity 1260 HPLC–UV system (Agilent Technologies, Santa Clara, CA, USA) equipped with OpenLab CDS ChemStation Edition C.01.10 software (Agilent Technologies) for data acquisition and analysis. The mobile phase consisted of 0.1% formic acid in water (A) and 0.1% formic acid in acetonitrile (B). Separation was carried out on a Thermo BDS Hypersil C18 semi-preparative column (250 × 10 mm i.d., 5 µm; Thermo Fisher Scientific, Waltham, MA, USA) using the following gradient elution: 0–15 min (50% B), 15–16 min (50–100% B), 16–20 min (100% B), 20–21 min (100–50% B), and 21–25 min (50% B). The flow rate was set at 5 mL min^−1^, and the injection volume was 100 µL. The column temperature was maintained at 25 °C. Chromatograms were recorded at 210 nm, 280 nm, and 340 nm. Using an Agilent 1260 Infinity II fraction collector, the fraction eluting between 12.3 min and 14.0 min was collected using a time-triggered event. After every 10 runs, the column was washed with 100% B for 10 min before resuming. A total of 284 consecutive runs were carried out. The collected fractions were pooled, the solvent removed under a stream of nitrogen, and the solid residue weighed (20 × 2^nd^D separations resulted in approximately 1.36 mg of erinacine A). A small amount of the isolated erinacine A was dissolved in 70% aqueous ethanol at a concentration of 25 µg mL^−1^ and filtered through a 0.2 µm PTFE filter (Chromofil, Macherey-Nagel, Düren, Germany) for characterisation by HPLC–UV–MS/MS and HPLC–CAD.

### 2.7. Characterisation of the Final Isolate by HPLC–UV–MS/MS

For the HPLC–UV–MS/MS analysis, the Accela 1250 system coupled with an LTQ Velos MS system (Thermo Fisher Scientific) was used, while data acquisition and processing were carried out using Thermo Xcalibur 2.1.0 software (Thermo Fisher Scientific). An optimised HPLC–MS method using a Kinetex C18 column (100 mm × 4.6 mm, 2.6 µm; Phenomenex) was employed with the following gradient: 0–8 min (40% B), 8–15 min (100% B), 15–16 min (100–40% B), and 16–20 min (40% B). The mobile phase, composed of 0.1% formic acid in water (A) and 0.1% formic acid in acetonitrile (B), was run at a flow rate of 1.5 mL min^−1^, with a sample injection equal to 10 µL. The column temperature was maintained at 40 °C. Chromatograms were recorded at 210 nm, 280 nm, and 340 nm, along with UV spectra (190–800 nm). Electrospray ionisation in negative mode (ESI-) was employed for the compound ionisation and acquisition of MS and MS/MS spectra. The MS tune parameters were set as follows: the vaporizer temperature was maintained at 450 °C, while the transfer capillary temperature was set to 275 °C. The flow rates for the sheath, auxiliary, and sweep gasses were 50 arbitrary units (a.u.), 5 a.u., and 5 a.u., respectively. The discharge current was fixed at 5 µA, with the S-lens radio frequency offset held at 69%. The MS spectra were recorded in the range of *m/z* 50–2000, and the MS/MS spectra in the range of *m/z* 130–500, with MS/MS performed using a collision energy of 35%. UV chromatograms, total ion chromatograms (TIC), as well as MS and MS/MS spectra, were acquired for the (i) 1^st^D fraction, (ii) 2^nd^D fraction, and (iii) the custom-made standard used as a qualitative reference material.

### 2.8. Chromatographic Purity Assessment of the Isolated Erinacine A Using HPLC–CAD

The isolated erinacine A, at a concentration of 25 µg mL^−1^ in 70% ethanol, was analysed for chromatographic purity using a HPLC system equipped with CAD (Vanquish, Thermo Scientific). Chromeleon 7.2 CDS software (Thermo Fisher Scientific) was used for data acquisition and processing. The same column and mobile phase, as described in the previous section, were employed. The gradient elution was as follows: 0–4.5 min (30–50% B), 4.5–13.5 min (50–60% B), 13.5–27.0 min (60–100% B), 27–27.01 min (100–30% B), and 27.01–35 min (30% B). The flow rate was set at 1 mL min^−1^, with an injection volume of 10 µL. The CAD temperature was set at 45 °C, with the method operating at a power function of 1. The column temperature was maintained at 40 °C. The peak area normalisation method was utilised for the chromatographic purity assessment of the isolated compound. Since the solution of the isolated erinacine A underwent filtration prior to HPLC analysis, a procedural blank from the filtration process was also injected [22]. This blank was used to subtract the background stemming from leaching interferences, which originated from plastic filtration materials such as plastic syringes, filters, and pipettes.

## 3. Results and Discussion

### 3.1. Overview of the Methodology

**Figure 1:**
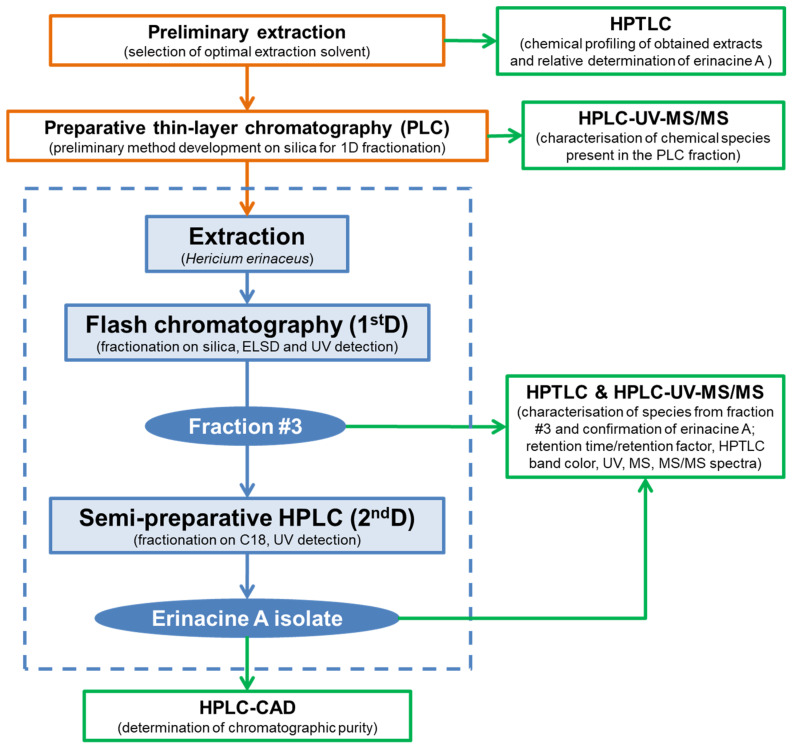
Schematic representation of the fractionation workflow: preliminary stage (orange), supporting analyses for isolation guidance and isolate characterisation (green), and the optimised isolation protocol (blue). The dashed blue frame highlights the key iterative steps that enabled the successful isolation of erinacine A.

A schematic representation of the entire isolation and purification workflow is illustrated in Figure 1, offering a simplified outline of key stages and supporting assays.

Sufficient solubility of erinacine A in a few solvents has been reported, with aqueous ethanol and ethyl acetate being the most common choices. Aqueous ethanol is generally used for the extraction of erinacine A, while ethyl acetate is typically employed during fractionation and purification [23]. The optimal extraction solvent for erinacine A from the *H. erinaceus* biomass was selected by screening four different solvents and solvent mixtures: ethanol, ethyl acetate, 70% ethanol(aq), and 70% ethyl acetate(aq). The HPTLC analysis showed the presence of erinacine A in all four extracts (Figure S1A–C). Pure organic solvents (ethanol and ethyl acetate) gave lower extraction yields of erinacine A compared to their aqueous mixtures. Although 70% ethanol did not yield the highest amount of material, it was favoured over 70% ethyl acetate, as it offered better extraction selectivity (fewer co-extracted compounds). Some of these co-extracted interfering compounds migrated closely to erinacine A (Figure S1B,C), which would have further complicated the subsequent fractionation process. Furthermore, 70% ethanol is environmentally friendly and generally recognised as safe (GRAS). It is already used as a safe ingredient in many commercial food and pharmaceutical products, making it a preferable choice for large-scale applications.

To preserve the fungal material, preparative silica-based TLC plates (PLC) were used for a preliminary small-scale fractionation of the 70% ethanolic extract of *H. erinaceus*. After developing the plate, the erinacine A-containing band was extracted from the PLC adsorbent (Figure S2) and analysed by HPLC–UV–MS/MS. The results revealed the presence of two closely eluting isobaric compounds (partially and fully separated before and after method optimisation, respectively; Figure S3A,B), not discernible by PLC. The MS spectrum of the early eluting chromatographic peak showed a strong signal at *m/z* 477 [M+HCOO]^−^ and a less intense signal at *m/z* 467, both of which were also present in the MS spectrum of the custom-made reference material (erinacine A). The later eluting compound showed the same adduct ion in the MS spectrum at *m/z* 477, but exhibited a different fragmentation pattern (Figure S3A,B). Given the similarity of the chromatographic retention and MS data, the later eluting compound was likely a related erinacine species that we wanted to avoid in the final isolate.

**Figure 2:**
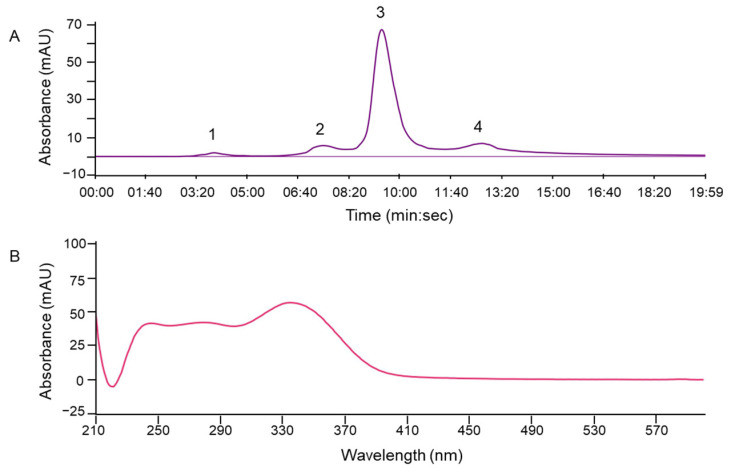
Flash–UV chromatogram at 340 nm showing fractions #1–4 (**A**) and UV–VIS spectrum of the erinacine A-containing fraction #3 (**B**).

**Figure 3:**
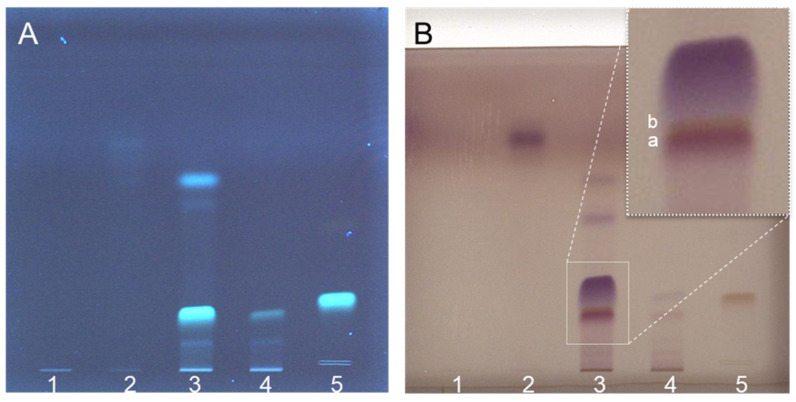
Chromatograms of fractions #1–4 (bands 1–4) and a custom-made erinacine A standard (band 5) using a HPTLC silica gel 60 plate. The images were acquired before (**A**) and after derivatization (**B**) with an anisaldehyde detection reagent at 366 nm (**A**) and under visible light (WhiteT) (**B**). A magnified view of track 3 (white dashed frame) reveals two partially co-eluting bands: a purple band with a lower retention factor (**a**) and a brownish band with a higher retention factor (**b**). The brownish band matched the colour of erinacine A.

**Figure 4:**
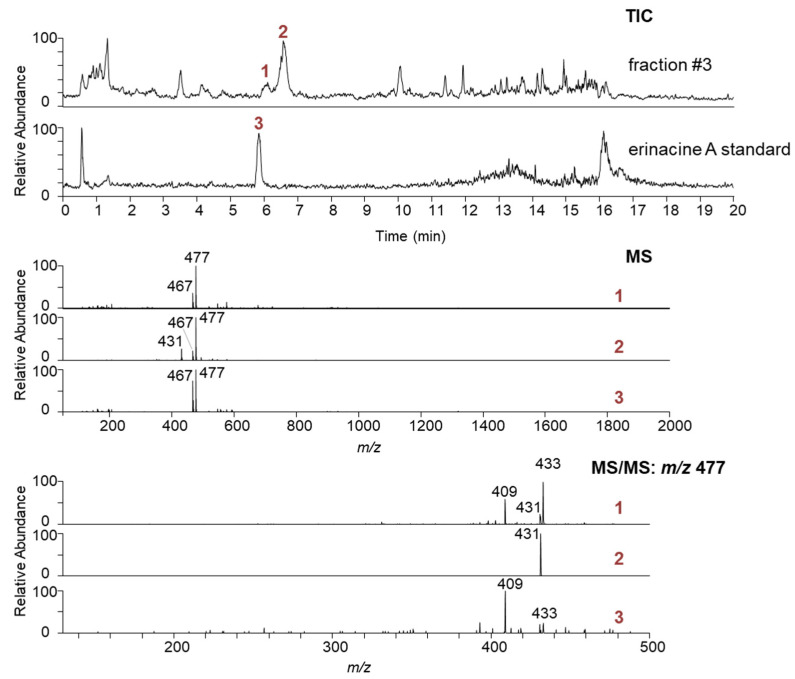
Total ion chromatogram (TIC) of the isolated fraction #3 from 1^st^D of fractionation (**top**); MS spectra of the closely eluting isobaric compounds (**middle**); and their MS/MS spectra (**bottom**). Data for the custom-made erinacine A standard are provided at the bottom of each section for reference.

The optimised PLC method was transferred to the flash chromatographic system used to fractionate the entire crude ethanolic extract on silica. Four distinct fractions (#1–4) were obtained (Figure 2A), with fraction #3 showing an absorbance maximum at 340 nm, characteristic of erinacine A (Figure 2B). The presence of erinacine A in fraction #3 was also confirmed by HPTLC (Figure 3A,B) and HPLC–UV–MS/MS (Figure 4). An intense HPTLC band at 366 nm was observed for fraction #3, which co-migrated with that of the erinacine A standard. Fraction #4 also showed a band at this retention factor (Figure 3A), but a mismatch of band colours after derivatisation of the plate clearly showed that this species was not erinacine A (Figure 3B inset). The presence of erinacine A in fraction #3 was further confirmed by the suitable retention times, MS, MS/MS (Figure 4), and UV–VIS data from the HPLC analysis.

**Figure 5:**
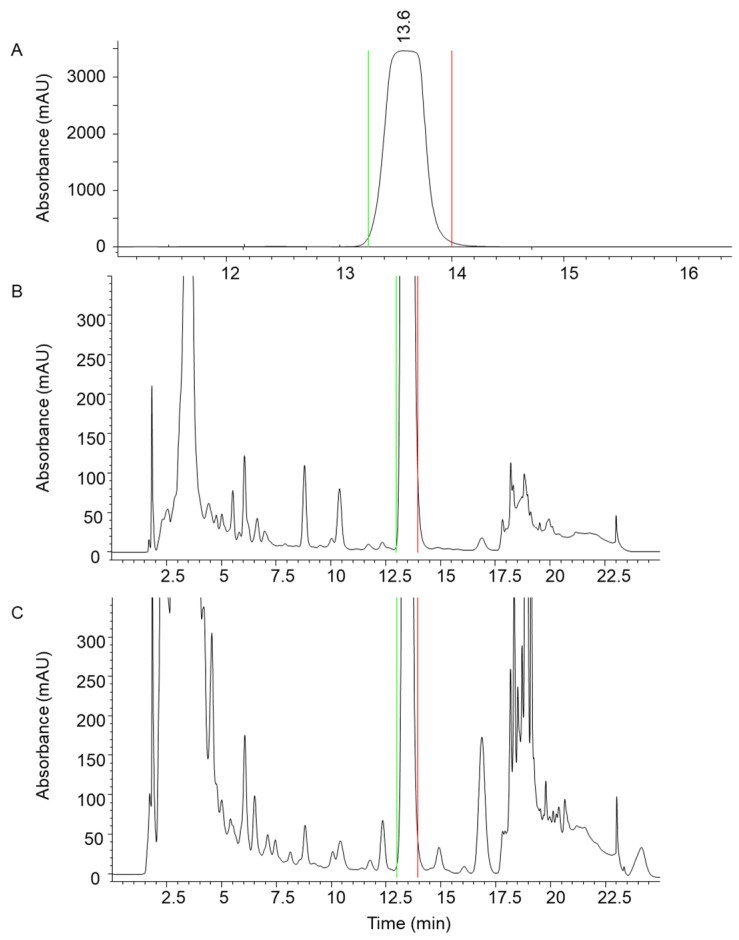
HPLC–UV chromatogram of fraction #3 at 340 nm (**A**) and a magnified view of the chromatogram at 340 nm (**B**) and 280 nm (**C**). The collection interval is indicated, with the green line marking the start and the red line marking the end of the collection.

For the final polishing step, a semi-preparative RP (C18) HPLC–UV method was used to further purify erinacine A from flash fraction #3 (Figure 5A) using the heart-cut chromatographic approach. Heart cutting is well-established in multidimensional chromatography, where only a single fraction from the first dimension is transferred to the second dimension for further separation of compounds [24].

**Figure 6:**
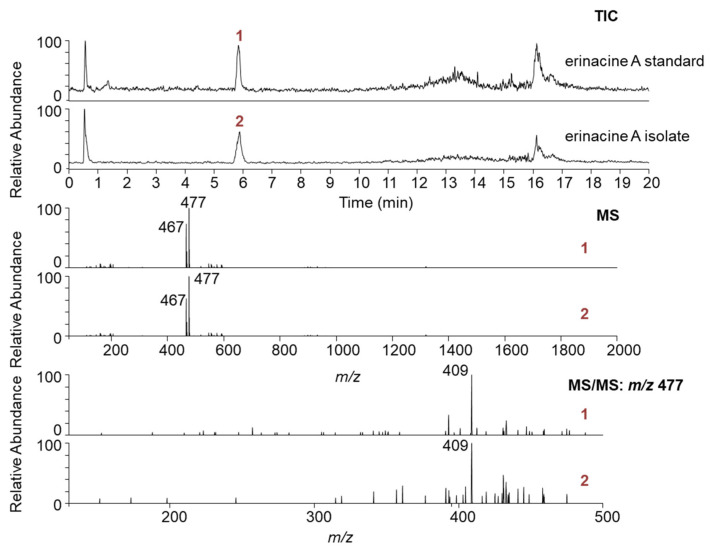
TIC (**top**), MS (**middle**), and MS/MS (**bottom**) spectra of the isolated erinacine A. Data for the custom-made erinacine A standard are provided at the top of each section for reference.

**Figure 7:**
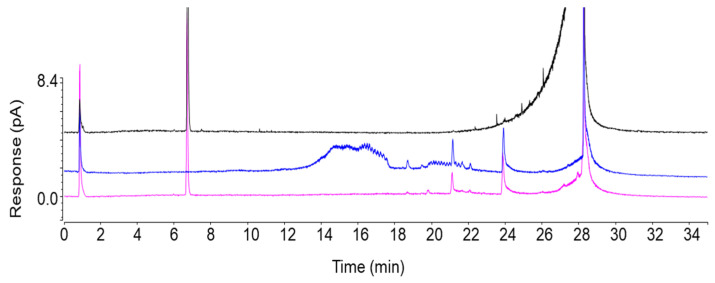
HPLC–CAD chromatograms of erinacine A standard (black), procedural blank (filtered 70% ethanol; blue), and the erinacine A isolate (pink).

The optimised method allowed for the satisfactory separation of erinacine A from all remaining interferences, including the closely eluting minor compounds (Figure 5B,C). After several repeated fractionation runs in both dimensions, 19.4 mg of erinacine A was isolated from approximately 130 g of fungal material. The TIC, MS, MS/MS (Figure 6), and UV–VIS data clearly confirmed the presence of erinacine A in the final isolate. The chromatographic purity of 97.4% was determined for erinacine A using HPLC–CAD (Figure 7). The area normalisation method used in this study for the purity determination of the erinacine A isolate effectively mitigated potential analytical bias that could have been introduced by the unknown purity of the reference standard material.

In summary, we have developed a simple and reliable two-step chromatographic protocol for the isolation of erinacine A from *H. erinaceus* biomass. The isolation of erinacine A is a difficult task due to the heavy matrix of the *H. erinaceus* biomass, which is further complicated by the presence of structurally similar erinacines and the added substrate in which erinacine A is also likely to be present [8,14]. However, the proposed 2D heart-cut chromatographic approach showed high specificity and separation efficiency.

### 3.2. Important Considerations

Preliminary optimisations and testing were critical to adapting the methodology to erinacine A. These included (i) screening for the most suitable solvent that selectively extracts erinacine A from the fungal material, thus reducing the complexity of the crude extract, (ii) optimising PLC separation as a preliminary isolation step that could be effectively scaled up and transferred to flash chromatography, and (iii) establishing a suitable 2D fractionation space characterised by orthogonality, enabling the production of erinacine A in pure form. NP flash chromatography, performed on silica with a larger particle size and higher capacity, allowed high sample loading and was therefore used to roughly remove the impurities from the crude extract of the *H. erinaceus*. However, a single fractionation step was not sufficient to resolve structurally similar and closely eluting impurities from erinacine A (Figure 4). The second, semi-preparative purification step using a high-performance RP stationary phase with 5 µm particles provided the additional selectivity and separation efficiency to remove the remaining impurities.

The erinacine A isolate was comprehensively characterised by complementary assays to increase the reliability of the compound’s annotation and to avoid false positive results. The chromatographic retention time of the isolate corresponded to that of the erinacine A reference standard using either of the RP chromatographic methods (HPLC–UV–MS/MS, semi-preparative HPLC–UV, or HPLC–CAD). While other erinacine derivatives have the same mass (e.g., erinacines B, E, and F) or are structurally closely related to erinacine A (erinacines C, D, G, H, I, J, K, P, Q, R, S, T, U, V, W, X, Y, Z1, and ZA), the multi-parameter comparison with the reference standard (HPLC retention time, HPTLC band colour and retention factor, UV–VIS, MS, and MS/MS spectra) allowed their differentiation and proper separation. At the time of writing, only a few erinacine analogues (e.g., erinacines A, B, and C) are commercially available. With the appropriate modifications, the proposed erinacine 2D fractionation platform could, therefore, also be used for the selective isolation of other bioactive species of the erinacine family.

To monitor the separation by flash chromatography, the ELSD detector, used in combination with the UV detector, provided a more comprehensive data set. The ELSD was essential for the detection of non-chromophoric impurities during the isolation of erinacine A in 1^st^D, which gave a strong signal at 340 nm by using the UV detector. This dual detection strategy facilitated the development of the flash chromatographic method. The CAD detector proved invaluable for assessing the purity of the isolated erinacine A in the absence of a certified reference material. The CAD detector is considered a universal detector due to its unique detection technology, which can detect potential impurities that are not necessarily observed by UV and/or MS detectors.

### 3.3. Comparison of the Proposed Isolation Methodology to Existing Approaches

The literature reports on the purification of erinacine A from *H. erinaceus* are almost exclusively based on open-column chromatography using bare silica as a stationary phase, e.g., [10,15,16,17,18]. The mycelium is generally treated with ethanol and the obtained extract is further partitioned between ethyl acetate and water. The organic phase is then chromatographed over silica gel using *n*-hexane-ethyl acetate step gradients. The solvent of the fraction containing erinacine A is evaporated to obtain a yellow amorphous powder, where the yellow colour likely indicates the presence of impurities. The pure erinacine A produced here was colourless, while the fraction #3 obtained after the first round of fractionation was pale yellow. Although open-column chromatography is characterised by its simplicity and speed, the reproducibility of such fractionation is usually poor due to the manual packing of columns, varying quality of silica, and possible variations in the sample preparation protocols. This can lead to insufficient erinacine A recovery and purity, which are normally not given in these reports. The 2D fractionation method proposed here, although more laborious, provides significant improvements in terms of efficiency, reliability, and automation of erinacine A isolation and characterisation. Such an approach is advantageous and justifiable when high-purity erinacine A is required.

Cheng et al., 2021, utilised the solid-state cultivation of *H. erinaceus* to produce and isolate erinacine A [11]. The downstream processing of the diterpenoid followed a modified open-column fractionation procedure, as described above, but on an approximately 200-fold smaller (analytical) scale. The fraction containing erinacine A was subjected to an additional round of purification on a semi-preparative C18 HPLC column. The yield and purity of the isolated erinacine A were unfortunately not reported, but signals in the MS spectrum indicated that other chemical species could also be present in the recovered material; for example, the signal at *m/z* 457 could be tentatively assigned to erinacine C, and so on. Higher molecular weight compounds could also be present, but their detection would be outside the defined MS scan range (*m/z* 400–500). In contrast, the isolation protocol described here provides a more comprehensive approach to the characterisation of the intermediate and final isolate by integrating both complementary qualitative and quantitative assays (HPTLC, HPLC–UV–MS/MS, HPLC–CAD).

Rupcic et al., 2018, isolated erinacine Z1 and Z2 as novel compounds from the crude acetone extract of *H. erinaceus* [25]. Erinacine A was also tentatively identified and isolated as an additional compound by using three sequential non-orthogonal RP fractionation steps. In the first step the crude extract was processed by flash chromatography on a C18 packed cartridge and eight fractions were obtained. The fraction containing erinacine A was then subjected to preparative HPLC fractionation on a Nucleodur C18 stationary phase. Due to the insufficient purity of erinacine A, the obtained fraction was purified additionally on a Kromasil C18 preparative HPLC column with multiple repetitions to finally afford 2.24 mg of erinacine A from 1.24 kg of mushroom material. Since the study was not focused on erinacine A but rather on the novel *H. erinaceus* compounds, it is difficult to compare the performance of the reported laborious isolation procedure with the fractionation platform presented here since no supporting information is provided regarding the purity of the isolated compound.

Only recently, an isolation of erinacine A by HSCCC was proposed by Liu et al., 2024 [9]. It is based on the partitioning of the compounds within a two-phase solvent system, leading to their partial or complete separation. The solvent system, consisting of *n*-hexane, ethyl acetate, methanol and water in a ratio of 4.5:5:4.5:5 (*v/v/v/v*), was used for the fractionation of the extract of *H. erinaceus* mycelia. The high purity (over 95%) of the isolated erinacine A was determined chromatographically by UV at 340 nm. However, our results clearly show that at this absorption wavelength, many impurities lacking a corresponding chromophore give a false negative result, leading to a potential overestimation of the purity of the compound (additional impurities visible in TIC-MS; Figure S4A,B). Some of the potential impurities can be detected at lower wavelengths with UV, but their detection is more reliable with a universal detector such as MS or CAD. At the same time, many erinacine A isomers and analogues present in *H. erinaceus* also absorb at 340 nm and elute closely to the target compound, making them difficult to distinguish without MS and MS/MS data. While HSCCC is more environmentally friendly due to minimal solvent waste and the absence of a stationary phase, flash chromatography and semi-preparative chromatography also have many clear advantages, such as the speed of analysis, simplicity, automation, and cost-effectiveness. The most important advantage of a heart-cut 2D chromatographic fractionation used here over HSCCC is probably the higher separation resolution, which has never been the particular strength of the CCC technique in general.

When accessible, detectors such as ELSD and CAD are very useful not only for assessing the chromatographic purity of the isolate but also for fine-tuning the chromatographic parameters during the development of the (fractionation) methods. Both ELSD and CAD exhibit good sensitivity and do not require specific chemical features or chemical derivatisation to generate measurable signals, ensuring accurate purity determination for a wide range of compounds. While the protocol proposed in this study offers significant advantages over known approaches, such as higher efficiency, robustness, and reliability, it is also important to recognise certain limitations. In particular, the protocol’s reliance on specialised instrumentation may reduce its utilisation. Moreover, the relatively low content of erinacine A in our starting material (approximately 1 mg per g) required many repeated fractionations to successfully accumulate the material at the milligram scale. This is not a drawback of the isolation platform per se but rather reflects the composition of the standardised food supplement, which contains a lower proportion (0.1%) of erinacine A compared to certain mushroom strains. Nevertheless, this makes the de novo elucidation of the isolated compound by nuclear magnetic resonance (NMR) spectroscopy and the determination of its absolute purity a formidable task. Strains with presumably higher levels of erinacine A facilitate faster acquisition of the isolate and enable ^1^H, ^13^C, heteronuclear correlation (HETCOR), and distortionless enhancement by polarisation transfer (DEPT) NMR analyses [10]. While NMR is a powerful tool for the structural confirmation of compounds, it lacks sensitivity and may be limited in detecting trace impurities, especially in a matrix that contains related erinacines. For absolute purity assessment, quantitative NMR (qNMR) could be employed, but it requires a reference standard of known purity for accurate quantification.

### 3.4. Preliminary Cost Analysis for Erinacine A Isolation

The isolation of 19.4 mg of erinacine A required approximately 18 L of Milli-Q water, 9 L of ethanol, 1.7 L of ethyl acetate, 28 L of acetonitrile (HPLC grade), and 70 mL of formic acid. At the time of writing, the total cost of silica-based cartridges for flash chromatography, which presents the main financial burden, and the solvents amounted to approximately EUR 3386 for 19.4 mg erinacine A, i.e., approximately EUR 175 per mg. These costs are significantly lower in comparison to most commercial products of similar purity (e.g., EUR 1322 (MedChemExpress), USD 850 (MedKoo Biosciences), EUR 1058 (Targetmol Chemicals), EUR 986 (Biosynth), EUR 2072 (TRC Canada)) or at least comparable (e.g., EUR 177 (Cayman Chemicals), and USD 154 (ChromaDex Standards)), although it is important to note that this comparison does not account for other associated costs (e.g., personnel, instrumentation, packaging, marketing, distribution, utilities, facility, etc.). Recycling solvents and reusing disposable silica flash cartridges could further reduce production costs and minimise waste generation while simultaneously maintaining purity and high throughput. However, further testing is needed to determine the actual savings. It should be noted that other important expenses such as electricity, equipment amortisation, and the cost of supporting analyses have not been included in the calculation for simplicity reasons, as the total cost is mainly set by the consumption of cartridges and acetonitrile. The above estimate provides some insight for those wishing to replicate the isolation process and serves as a basis for further economic evaluations, particularly for larger-scale applications that typically require detailed life cycle assessments.

## 4. Conclusions

In this study, we developed a two-dimensional fractionation protocol for the isolation of erinacine A from *H. erinaceus* biomass. Following the initial optimisation of the protocol, our demonstrative procedure, using an efficient 2D combination of NP and RP purification, yielded 19.4 mg of erinacine A from approximately 130 g of *H. erinaceus* raw material and achieved a chromatographic purity of 97.4%. The integration of orthogonal monitoring assays enabled the identification of erinacine A with greater confidence and guided the purification process. The use of HPLC–CAD was crucial in estimating the purity of the isolate, as sole reliance on UV-based detection of impurities can lead to biased results. Considering that commercial erinacine A standards are scarce, the isolation platform serves as an important resource for research laboratories wishing to produce their own erinacine A standard with low impurity content. The chromatographic methods developed here can also readily be used for the quality control of *H. erinaceus* feedstock and its extracts. Beyond cost-effectiveness, it should be emphasised that the proposed isolation method also enables the potential recovery of other bioactive compounds of the erinacine family, as it allows discrimination between different isomers by fine-tuning the chromatographic conditions. In contrast to the most studied erinacine A compound, other erinacine derivatives are mostly not commercially available. This contribution not only facilitates further research and clinical trials of this neuroprotective compound but also enables the use of erinacine A in therapeutic applications, paving the way for future studies on its efficacy in the treatment of neurodegenerative diseases.

## Data Availability

The raw data supporting the conclusions of this article will be made available by the authors upon request.

## Supplementary Materials

The following supporting information can be downloaded at: https://www.mdpi.com/article/10.3390/jof11020150/s1, Figure S1: Comparison of *Hericium erinaceus* biomass extracts (bands 1–4) with each other and in reference to a custom-made erinacine A standard (band 5) using a HPTLC silica gel 60 plate. The extracts were prepared using various solvents and solvent mixtures as follows: ethanol (1), 70% ethyl acetate (2), ethyl acetate (3), and 70% ethanol (4). The plate was captured before (A) and after derivatization (B,C) with the anisaldehyde detection reagent at 366 nm (A), under visible light (WhiteT) (B) and at 254 nm; Figure S2: Fractionation of 70% ethanol extract (900 µL per plate, 180 mm band) using a PLC silica gel 60 plate. The edges of the plate were derivatized with the anisaldehyde detection reagent. The underivatized plate part, corresponding to erinacine A standard (circled), was scratched off; Figure S3: EIC at *m/z* 477 (top) and MS and MS/MS spectra (bottom left and right, respectively) of the isolated PLC fraction before (A) and after (B) HPLC–UV–MS/MS method optimization. Erinacine A standard was used for reference (B); Figure S4: HPLC–UV chromatograms at 340 nm (top), 280 nm (middle), and TIC (bottom) of fraction 3 (A); MS spectra of the closely eluting peaks observed in the TIC (B).
