# Supplementary material for: Two-Dimensional Chromatographic Isolation of High Purity Erinacine A from Hericium erinaceus
Source: J Fungi (Basel). 2025 Feb 15;11(2):150. doi: 10.3390/jof11020150 (PMC11856814; doi:10.3390/jof11020150)
Supplement: Supplementary file 1 [file jof-11-00150-s001.zip › jof-3383819-supplementary.pdf]

Supplementary information

Two-Dimensional Chromatographic Isolation of High Purity Erinacine A from *Hericium erinaceus*

Katerina Naumoska [1], Andrej Gregori [2,3] and Alen Albreht [1,*]

[1] Laboratory for Food Chemistry, Department of Analytical Chemistry, National Institute of Chemistry, Hajdrihova ulica 19, 1001 Ljubljana, Slovenia
[2] Mycomedica Ltd., Podkoren 72, 4280 Kranjska Gora, Slovenia
[3] Biotechnical Faculty, University of Ljubljana, Jamnikarjeva ulica 101, 1000 Ljubljana, Slovenia
* Correspondence: alen.albreht@ki.si; Tel.: +386-14760269

List of Figures:

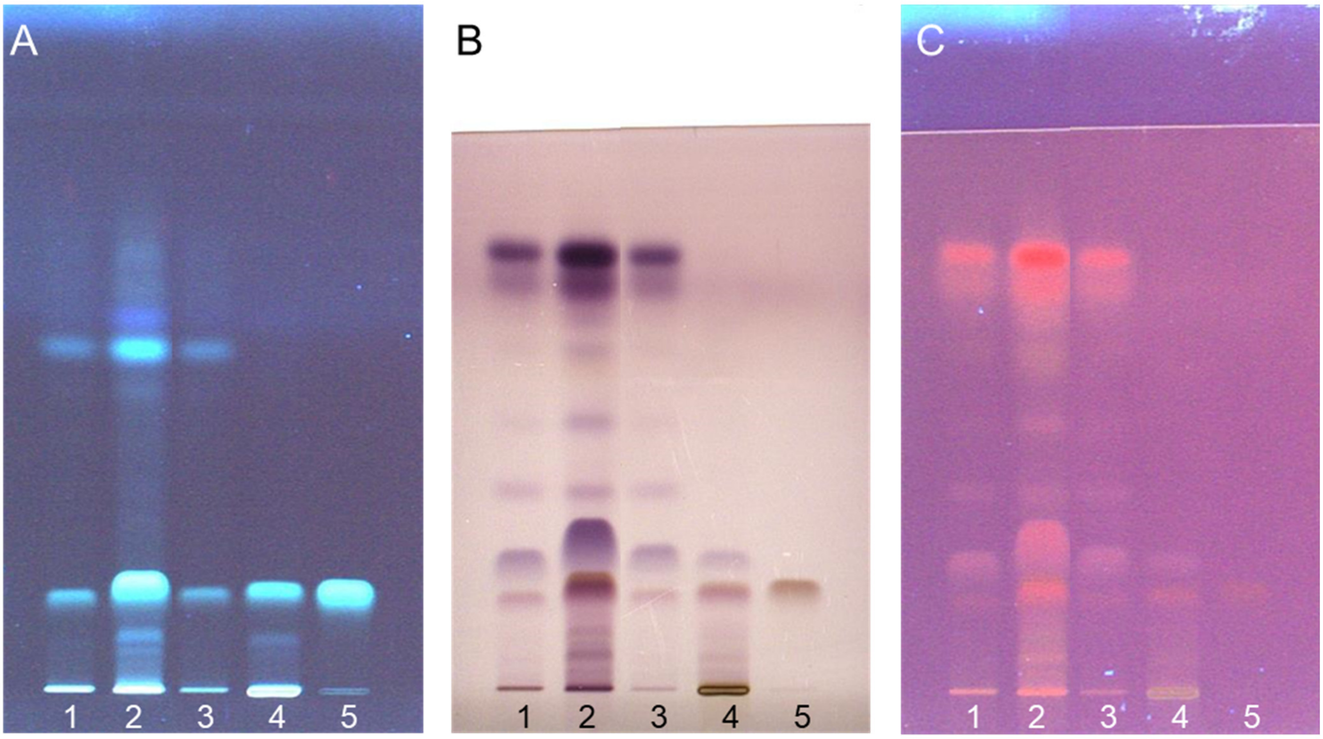

Figure S1. Comparison of *Hericium erinaceus* biomass extracts (bands 1–4) with each other and in reference to a custom-made erinacine A standard (band 5) using a HPTLC silica gel 60 plate. The extracts were prepared using various solvents and solvent mixtures as follows: ethanol (1), 70% ethyl acetate (2), ethyl acetate (3), and 70% ethanol (4). The plate was captured before (**A**) and after derivatization (**B**, **C**) with the anisaldehyde detection reagent at 366 nm (**A**), under visible light (WhiteT) (**B**) and at 254 nm (**C**).

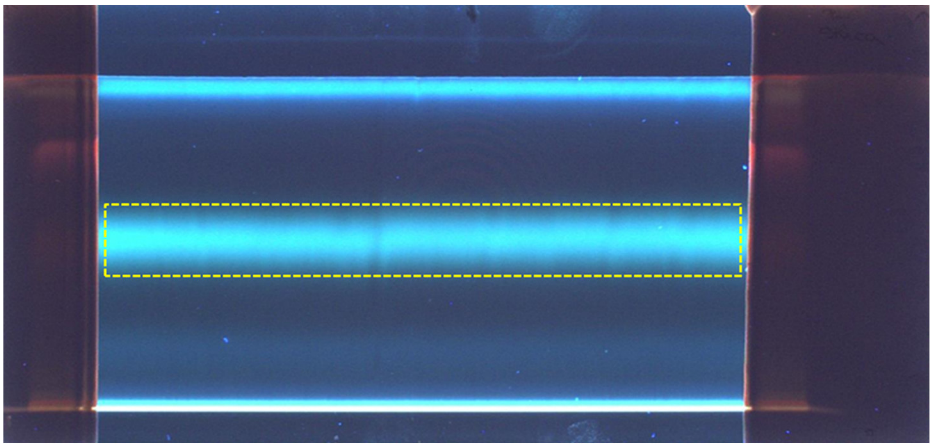

Figure S2. Fractionation of 70% ethanol extract (900 µL per plate, 180 mm band) using PLC silica gel 60 plate. The edges of the plate were derivatized with anisaldehyde detection reagent. The underivatized plate part, corresponding to erinacine A standard (circled) was scratched off.

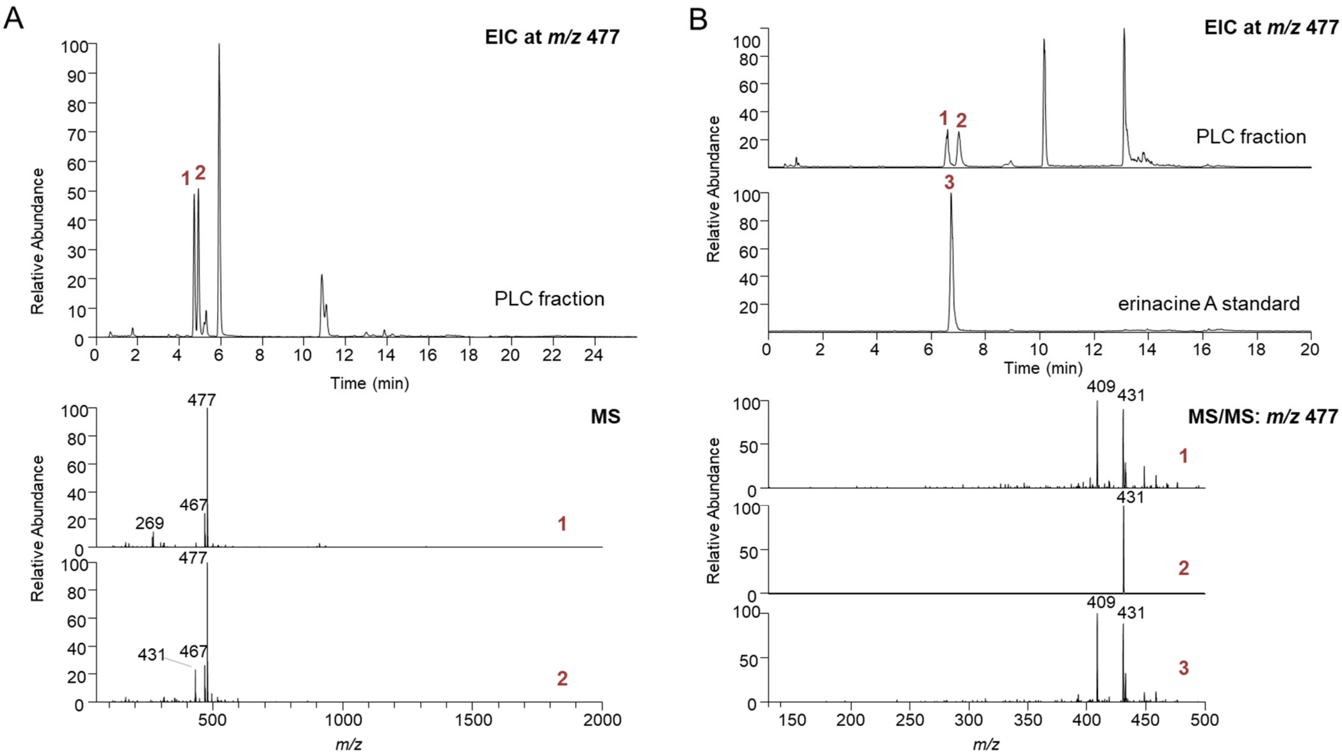

Figure S3. EIC at *m/z* 477 (top) and MS and MS/MS spectra (bottom left and right, respectively) of the isolated PLC fraction before (**A**) and after (**B**) HPLC—UV—MS/MS method optimization. Erinacine A standard was used for reference (B).

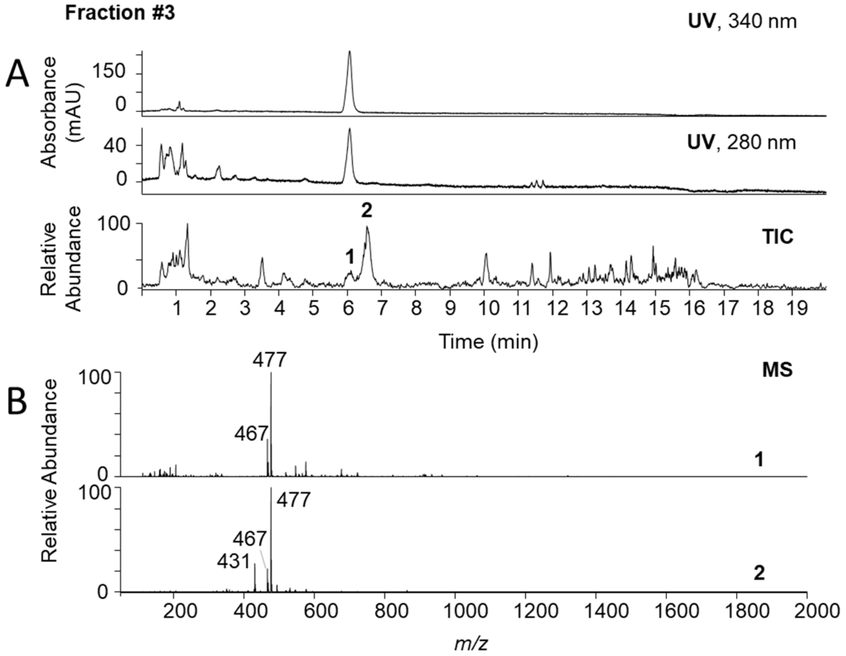

Figure S4. HPLC–UV chromatograms at 340 nm (top), 280 nm (middle), and TIC (bottom) of fraction 3 (A); MS spectra of the closely eluting peaks observed in the TIC (B).
